# Biphasic, Refractory, and Persistent Anaphylaxis in Children

**DOI:** 10.1002/clt2.70174

**Published:** 2026-05-05

**Authors:** Gizem Koken, H. Ilbilge Ertoy Karagol, Sinem Polat Terece, Ceren Varer Akpinar, Kenan Cetin, Zeynep Cavdar, Berkehan Kara, A. Kubra Baskin, Arzu Bakirtas

**Affiliations:** ^1^ Department of Pediatric Allergy and Immunology Gazi University Faculty of Medicine Ankara Turkey; ^2^ Department of Public Health Giresun University Faculty of Medicine Giresun Turkey; ^3^ Gazi University Faculty of Medicine Ankara Turkey

**Keywords:** anaphylaxis, biphasic, children, drug, food, persistent, phenotype, refractory, venom

## Abstract

**Background:**

A Delphi consensus report refined anaphylaxis phenotypes as biphasic, refractory, and persistent anaphylaxis (BA, RA, and PA). To date, no study in either pediatric or adult populations has comprehensively evaluated the full spectrum of anaphylaxis phenotypes as outlined in this consensus. The primary aim of this study was to identify these phenotypes and compare them with conventional anaphylaxis in children.

**Methods:**

Patients aged ≤ 18 years who were diagnosed with or followed up for anaphylaxis at our department over the past 15 years were retrospectively screened for this study. All anaphylaxis cases were categorized as conventional anaphylaxis (Group 1) or as BA, RA, and PA phenotypes (Group 2). A comparative analysis was conducted between Group 1 and Group 2 with respect to demographics, triggers, clinical features, severity, management, and outcomes.

**Results:**

A total of 393 patients and 529 anaphylaxis episodes were included. Twenty‐six (6.6%) of all anaphylaxis cases were classified as BA (3.5%), RA (1.5%), or PA (1.5%). For BA, the median time to recurrence of symptoms and signs was 4 h (1–24 h), whereas the median duration of PA manifestations was 4 h (4–6 h). These phenotypes (Group 2) were more common in older children and were associated with increased cardiovascular manifestations, greater severity, and higher use of systemic corticosteroid (*p* < 0.001). They did not differ significantly from Group 1 with respect to gender, comorbidities, family history of atopy, timing or location of the anaphylaxis, or number of episodes. Drugs, followed by venoms, were more frequent triggers in Group 2, whereas food was significantly more common in Group 1 (*p* < 0.05). IM adrenaline was administered in 69.2% of Group 2 and 52.3% of Group 1, with no significant difference (*p* > 0.05). Comparisons among BA, RA, and PA could not be performed due to the limited sample size within each phenotype.

**Conclusions:**

BA, RA, and PA are rare anaphylaxis phenotypes, more frequently drug or venom induced, seen at older ages, with ongoing gaps in proper IM adrenaline use.

## Graphical abstract

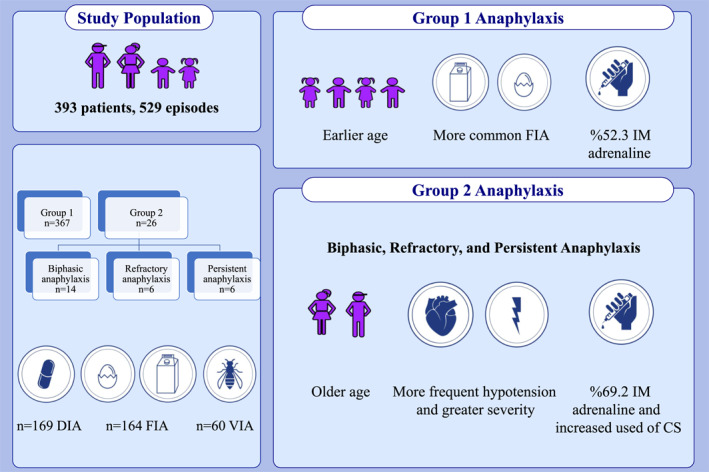

**Abbreviations**
BA: biphasic anaphylaxis
PA: persistent anaphylaxis
RA: refractory anaphylaxis

## 1 Introduction

Anaphylaxis is the most severe, potentially life‐threatening clinical presentation of acute systemic allergic reactions [1, 2, 3]. It is typically a multisystem reaction triggered by IgE‐mediated activation of mast cells and/or basophils in response to allergen exposure, commonly from food, drug, or venom, while in drug induced anaphylaxis, non‐IgE‐mediated mechanisms may also lead to anaphylaxis [4, 5, 6]. However, the etiology of anaphylaxis can sometimes be undetermined (idiopathic anaphylaxis), or it may arise due to mast cell disorders [1, 2].

Anaphylaxis requires immediate medical attention, with diagnosis predominantly based on clinical evaluation [1, 2, 7]. Beyond the accurate definition of anaphylaxis, there has been a growing need for new nomenclature to support proper management during treatment and follow‐up. In November 2020, a multidisciplinary Delphi consensus report introduced refined anaphylaxis phenotypes—biphasic, refractory, and persistent anaphylaxis—to assist healthcare professionals in improving management strategies [8].

Since then, three studies have focused on anaphylaxis in children, specifically examining biphasic and refractory anaphylaxis separately [9, 10, 11, 12]. However, to date, only one study has mentioned these anaphylaxis phenotypes [12]. This highlights the need for further research to better understand these new definitions, as they significantly impact the management of anaphylaxis [13]. To address this gap, we primarily aimed to evaluate the frequency and characteristics of biphasic (BA), refractory (RA), and persistent anaphylaxis (PA) in children and to compare these phenotypes with conventional anaphylaxis.

## 2 Methods

### 2.1 Study Design and Retrospective Record Review

Patients aged ≤ 18 years who were diagnosed with anaphylaxis at our institution or referred from other healthcare facilities with a diagnosis of anaphylaxis and subsequently followed in our department, between January 2010 and January 2025 were retrospectively screened for inclusion in this study. Ethical approval was obtained from the institutional ethics committee (2024‐1018).

The hospital's electronic database was systematically screened by GK using ICD‐10 codes corresponding to anaphylaxis, including T78.0 (anaphylactic reaction due to food), T78.2 (anaphylactic shock, unspecified), and T88.6 (anaphylactic shock due to the adverse effect of a correctly administered drug or medicament). Independent verification was conducted by HIEK and AB in accordance with established diagnostic criteria for anaphylaxis [14].

### 2.2 Diagnosis of Anaphylaxis

The diagnosis of anaphylaxis was established clinically based on the 2006 National Institute of Allergy and Infectious Disease/Food Allergy and Anaphylaxis Network (NIAID/FAAN) criteria [14], relying on acute‐onset objective clinical manifestations [15], including urticaria or angioedema accompanied by wheezing, vomiting, syncope, collapse, or incontinence, as well as physical examination findings such as rhonchi, hypoxia, and hypotension.

### 2.3 Severity of Anaphylaxis

The severity of anaphylaxis was classified into three groups: mild, moderate, and severe, based on the Brown's criteria [16].

### 2.4 Selection and Characterization of Anaphylaxis Cases

Anaphylaxis cases triggered by rare causes, mast cell disorders, and idiopathic anaphylaxis were excluded due to their low frequency in the study population. Patients with anaphylaxis induced by food, drugs, or venom were included in the final cohort. For each patient, demographic characteristics, triggers, clinical manifestations, severity, phenotypes (conventional anaphylaxis, BA, RA, and PA), management, and outcomes were systematically extracted from medical records.

To avoid duplicate patient‐level observations, recurrent episodes other than the index episode were excluded. In patients with a history of anaphylaxis associated with multiple triggers, the trigger identified at the index episode was used for analysis.

### 2.5 Diagnostic Evaluation and Confirmation of Anaphylaxis Triggers

All patients with food induced anaphylaxis (FIA) and venom induced anaphylaxis (VIA) were initially diagnosed based on clinical impressions documented in the medical records, followed by confirmation through skin testing and/or specific IgE measurements. In patients with drug induced anaphylaxis (DIA), the diagnosis was established based on clinical documentation and, when clinically appropriate, confirmed by skin tests and drug provocation tests. However, in some cases, only a clinical diagnosis could be made due to factors such as a history of severe anaphylaxis, ongoing treatment, or the unavailability or unsuitability of the suspected drug for testing. In cases with diagnostic uncertainty or competing triggers, the diagnosis was based on the most likely trigger as determined by clinical evaluation and the available diagnostic work‐up.

Serum tryptase levels, both during the reaction and at baseline, were also evaluated in cases for which such data were available based on review of the medical records.

### 2.6 Definitions

#### 2.6.1 Biphasic Anaphylaxis (BA)

The recurrence of anaphylaxis symptoms and signs at least 1 h, and up to 48 h, after the complete resolution of the initial anaphylaxis symptoms and signs, without re‐exposure to the allergen [8].

Time zero for BA was defined as the point at which the initial manifestations of anaphylaxis had completely resolved. The median time to BA was calculated as the interval in hours from time zero to the reappearance of symptoms and signs.

#### 2.6.2 Refractory Anaphylaxis (RA)

The continuation of symptoms and signs of the initial anaphylaxis despite two or more appropriate doses of intramuscular adrenaline (or adrenaline infusion) and symptomatic treatment (such as intravenous hydration and oxygen support) [8, 17, 18].

#### 2.6.3 Persistent Anaphylaxis (PA)

The persistence of anaphylaxis symptoms and signs for at least 4 h [8].

Time zero was defined as the onset of anaphylaxis manifestations, and the duration of PA was defined as the interval from time zero to complete resolution.

### 2.7 Comparison of Anaphylaxis Cases

Anaphylaxis cases that did not meet the criteria for biphasic, refractory, or persistent anaphylaxis phenotypes were classified as Group 1, while those exhibiting any of these phenotypes were classified as Group 2. A comparative analysis was planned to examine differences both among BA, RA, and PA phenotypes and between these phenotypes (Group 2) and Group 1 with respect to demographics (sex, age, chronic comorbidities, and family history of atopy), as well as triggers, anaphylaxis setting, time to onset, clinical features, severity, management, and outcomes.

### 2.8 Statistical Analysis

The data were analyzed using SPSS version 25.0 statistical software. Descriptive statistics were presented as numbers and percentages for categorical variables, and as median with interquartile range, or minimum and maximum values for numerical variables. The chi‐square test was used to compare categorical variables. For comparing numerical variables, normal distribution assumptions were evaluated using Kolmogorov–Smirnov and Shapiro–Wilk tests, followed by the Kruskal–Wallis test. A two‐sided *p* value of less than 0.05 was considered statistically significant.

## 3 Results

**FIGURE 1:**
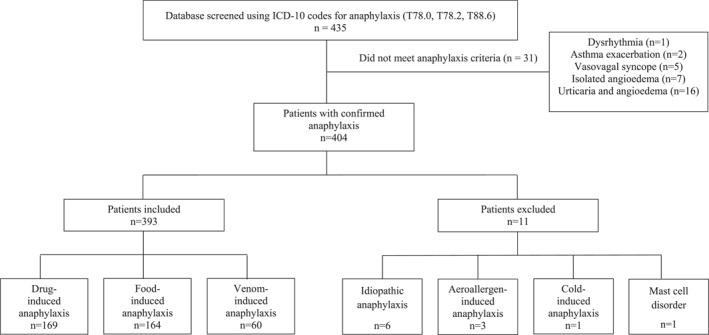
Flowchart of patient selection.

The database was screened using ICD‐10 codes for anaphylaxis, identifying 435 patients; 31 were not eligible as they did not meet the diagnostic criteria for anaphylaxis. A total of 404 patients with confirmed anaphylaxis were identified. The estimated incidence of anaphylaxis was 142.4 per 100,000 institutional pediatric emergency department visits. After the exclusion of 11 patients due to rare triggers or mast cell disorders, the final cohort comprised 393 patients who experienced a total of 529 anaphylaxis episodes (Figure 1). Overall, 61.6% of the patients were male, and the median age was 49 months (IQR: 112.5 months).

According to triggers, 169 patients had drug‐induced anaphylaxis (DIA) (43%), 164 had food induced anaphylaxis (FIA) (41.7%), and 60 had venom induced anaphylaxis (VIA) (15.3%). Five patients (1.3%) had experienced anaphylaxis with two different triggers (drug and food: 3 patients, drug and venom: 2 patients).

In the overall cohort, 142 anaphylaxis cases occurred within medical facilities, whereas 251 occurred in community settings. Among patients with DIA, 31 (18.3%) experienced anaphylaxis at home and 138 (81.7%) within a medical facility. All patients with VIA and 160 patients (97.6%) with FIA experienced anaphylaxis in community settings. Only four patients with FIA developed anaphylaxis in the hospital during oral provocation testing.

Diagnostic testing was performed in all patients with FIA and VIA and, when clinically appropriate, in patients with DIA. Some patients with DIA, only a clinical diagnosis could be established due to factors such as a history of severe anaphylaxis, ongoing treatment or unavailability or unsuitability of the suspected drug for testing. Serum tryptase measurements were not available for all patients; therefore, a comprehensive evaluation of tryptase levels was not feasible.

A total of 367 patients (93.4%) were classified as Group 1 (conventional anaphylaxis), while 26 patients (6.6%) were classified as Group 2, including 14 patients with BA (3.6%), six with RA (1.5%), and six with PA (1.5%).

### 3.1 Biphasic, Refractory, and Persistent Anapylaxis

The baseline characteristics of BA, RA, and PA are presented in Table 1. Drugs were the most common anaphylaxis triggers across all three phenotypes. Venoms triggered anaphylaxis in half of the PA cases and one‐third of the RA cases. Food was identified as a trigger only in cases of BA.

**TABLE 1:** Baseline characteristics of biphasic, refractory, and persistent anaphylaxis.

	Biphasic anaphylaxis (*n* = 14)	Refractory anaphylaxis (*n* = 6)	Persistent anaphylaxis (*n* = 6)
Male, *n* (%)	7 (50.0)	3 (50.0)	5 (83.3)
Age at anaphylaxis, months	76 (IQR: 136)	142.5 (IQR: 78.2)	68.5 (IQR: 75)
Atopic comorbidity, *n* (%)			
Asthma	5 (35.7)	0 (0)	0 (0)
Atopic dermatitis	3 (21.4)	0 (0)	0 (0)
Allergic rhinitis	2 (14.3)	0 (0)	0 (0)
Trigger, *n* (%)			
Drug	8 (57.1)	4 (66.7)	3 (50.0)
Food	4 (28.6)	0 (0)	0 (0)
Venom	2 (14.3)	2 (33.3)	3 (50.0)
Location of anaphylaxis, *n* (%)			
Inside medical facility	6 (42.9)	4 (66.7)	2 (33.3)
Outside medical facility	8 (57.1)	2 (33.3)	4 (66.7)
Time to anaphylaxis, minutes	20 (1–60)	12.5 (5–30)	5 (1–120)
Severe anaphylaxis signs, *n* (%)			
Hypoxia	2 (14.3)	2 (33.3)	2 (33.3)
Bronchospasm	1 (7.1)	0 (0)	2 (33.3)
Laryngospasm	1 (7.1)	2 (33.3)	2 (33.3)
Hypotension	3 (21.4)	6 (100.0)	5 (83.3)
Severity, *n* (%)			
Severe	5 (35.7)	6 (100.0)	6 (100.0)
Moderate	8 (57.1)	0 (0)	0 (0)
Mild	1 (7.1)	0 (0)	0 (0)
Management, *n* (%)			
No adrenaline	6 (42.8)	0 (0)	1 (16.6)
One IM adrenaline	8 (57.1)	0 (0)	0 (0)
≥ 2 IM adrenaline (without infusion)	0 (0)	1 (16.7)	5 (83.3)
Adrenaline infusion	0 (0)	5 (83.3)	0 (0)
Antihistamine	11 (78.6)	2 (33.3)	5 (83.3)
Systemic corticosteroid	12 (85.7)	3 (50.0)	5 (83.3)
Number of episodes	1 (1–3)	1 (1–2)	1

*Note:* Continuous variables presented as median (interquartile range) or median (min–max).

The median time to BA was 4 h (1–24 h). Cases with BA initially presented with mild (*n* = 1), moderate (*n* = 8), and severe (*n* = 5) grades. During the second reaction of BA, severity was mild in 5 cases, moderate in 8, and severe in 1. All patients diagnosed with RA or PA experienced severe anaphylaxis. The median duration of PA was 4 h (4–6 h). Intramuscular (IM) adrenaline treatment was not administered to 42.8% of patients in initial reaction of BA. Adrenaline infusion was administered in five RA cases (83.3%), while one patient received three intramuscular doses without requiring infusion. One patient with drug induced RA died due to hypotension unresponsive to inotropic treatments. There were no patients using beta‐blockers in any of the RA or PA cases. The use of antihistamines and systemic corticosteroids (CS) was higher in patients diagnosed with BA and PA (Table 1).

Comparative analyses among the BA, RA, and PA phenotypes could not be performed due to the limited sample size within each phenotype.

### 3.2 Comparison of Anaphylaxis Groups

Groups were compared based on gender, age at anaphylaxis, allergic and non‐allergic chronic comorbidities, family history of atopy, as well as triggers, location of anaphylaxis, time to symptom onset, clinical manifestations, severity, management, and number of episodes (Table 2).

**TABLE 2:** Comparison of anaphylaxis based on phenotypes.

	Group 1 *n* = 367	Group 2 *n* = 26
Male, *n* (%)	227 (61.9)	15 (57.7)
Age at anaphylaxis, months	45 (IQR: 110)^*^	104 (IQR: 105)
Allergic comorbidity, *n* (%)	178 (48.5)	9 (34.6)
Non‐allergic chronic comorbidity, *n* (%)	97 (26.4)	7 (26.9)
Family history of atopy, *n* (%)	158 (43.1)	10 (38.5)
Trigger, *n* (%)		
Drug	154 (42.0)	15 (57.7)
Food	160 (43.6)^*^	4 (15.4)
Venom	53 (14.4)	7 (26.9)
Location of anaphylaxis, *n* (%)		
Inside medical facility	130 (35.4)	12 (46.2)
Outside medical facility	237 (64.6)	14 (53.8)
Time to anaphylaxis, minutes	10 (1–120)	12.5 (1–120)
Clinical manifestions, *n* (%)		
Cutaneous	338 (92.1)	26 (100.0)
Respiratory	282 (76.8)	23 (88.5)
Gastrointestinal	162 (44.1)	7 (26.9)
Cardiovascular	80 (21.8)	15 (57.7)^†^
Neurological	28 (7.6)	3 (11.5)
Severity, *n* (%)		
Severe	101 (27.5)	17 (65.4)^†^
Moderate	217 (59.1)	8 (30.8)
Mild	49 (13.4)	1 (3.8)
Management, *n* (%)		
IM adrenaline	192 (52.3)	18 (69.2)
Antihistamine	200 (54.5)	18 (69.2)
Systemic corticosteroid	144 (39.2)	20 (76.9)^†^
Adrenaline infusion	0 (0)	5 (19.2)
Number of episodes	1 (1–8)	1 (1–3)

*Note:* Continuous variables presented as median (interquartile range) or median (min–max).

^*^ *p* value < 0.05.

^†^ *p* value < 0.001 compared to the other group.

Group 1 anaphylaxis occurs at an earlier age (*p* = 0.02), while there is no significant difference between the two groups in terms of gender, allergic and non‐allergic chronic comorbidities, or family history of atopy (*p* > 0.05).

Food induced anaphylaxis was more frequently observed in Group 1 (*p* = 0.005), while there were no significant differences in the frequencies of DIA and VIA between the two groups (*p* > 0.05). No significant differences were observed in the location of anaphylaxis, time to symptom onset, or number of anaphylaxis episodes (*p* > 0.05).

Cardiovascular manifestations, anaphylaxis severity, and the use of systemic CS were significantly higher in Group 2 (*p* < 0.001). IM adrenaline was administered in 52.3% of Group 1 cases and 69.2% of Group 2 cases, with no statistically significant difference between the groups (*p* > 0.05).

Multivariable analyses were not feasible due to the limited number of cases in Group 2.

## 4 Discussion

There are only a limited number of studies that have evaluated anaphylaxis phenotypes: BA, RA, and PA separately in children [9, 10, 11, 12]. To date, no study has conducted a direct, head‐to‐head comparison that addresses all three phenotypes and compares them with conventional anaphylaxis phenotypes in either children or adults. To our knowledge, this is the first study to provide a detailed evaluation and comparison of these anaphylaxis phenotypes in a pediatric population. Fortunately, only a small proportion of children with anaphylaxis presented with BA, RA, or PA. These three phenotypes differ from conventional anaphylaxis cases in terms of age at anaphylaxis, triggers, clinical manifestations, severity, and management.

In our cohort, although the overall incidence of pediatric anaphylaxis was relatively high, these phenotypes were relatively uncommon. The incidence of pediatric anaphylaxis observed in our cohort appears consistent with rates reported in other hospital‐based studies in the literature [17, 18], although it may not fully reflect the true incidence, as our cohort also included patients referred from other healthcare facilities.

In a single study examining the effects of age and atopic status on pediatric anaphylaxis, the frequencies of RA, BA, and PA were reported as 8%, 2%, and 1%, respectively [12]. However, the study did not present any additional data or comparative analysis between these or other cases. While the frequencies of phenotypes other than RA were reported at comparable rates, the study reported a strikingly elevated prevalence of RA, for which no underlying explanation was provided [12]. In the European Anaphylaxis Registry, RA was rare, with a reported prevalence of 0.37%, defined by the need for ≥ 2 doses of IM adrenaline [19]. By comparison, a meta‐analysis found that approximately 7%–10% of anaphylaxis episodes required ≥ 2 doses of IM adrenaline, while about 2.2% required ≥ 3 doses [20]. Our study, conducted exclusively in pediatric cases, defined RA as anaphylaxis requiring ≥ 2 doses of IM adrenaline; had the definition been restricted to ≥ 3 doses, the prevalence would have been lower, from 1.5% to 1.1%.

Among these phenotypes, BA had more data reporting the frequency ranging from 0.3% to 5.1%, which were consistent with our results [10, 11, 12]. In our study, although we were unable to provide information regarding the timing of IM adrenaline, it was not administered during the index reaction in approximately half of the patients with BA. Therefore, we think that this is the principal cause of biphasic reactions.

Previous pediatric cohort studies have consistently demonstrated that exposure settings differ according to trigger type, with DIAs more frequently occurring in healthcare facilities, whereas FIA and VIAs predominantly arise in community settings [21, 22, 23]. Nevertheless, DIA appears to be overrepresented across all phenotypes in our study population, likely reflecting the tertiary referral nature of our center and the higher proportion of hospital‐acquired (iatrogenic) cases managed in this setting. Additionally, the European Anaphylaxis Registry (EAR) report and subsequent studies have demonstrated that RA is significantly more common in medical settings, particularly in the perioperative context, with drugs identified as the most frequent triggers [9, 19, 24]. In our study, no difference was observed between BA, RA, and PA phenotypes and conventional anaphylaxis cases with respect to the location of anaphylaxis, and perioperative anaphylaxis was not observed in any of the BA, RA, or PA cases.

In terms of age at index reaction, BA, RA, and PA phenotypes observed in relatively older children compared to conventional anaphylaxis. This may be explained by the higher prevalence of drugs and venoms as triggers in these phenotypes, which are typically encountered at older ages. On the other hand, food as more significant trigger in conventional anaphylaxis may be explained the younger age at presentation of this group.

In the study by Poussel et al. [9], children with RA were less likely to have a history of asthma or prior anaphylaxis. The EAR report also indicated that patients with RA more frequently had concomitant asthma, malignant diseases, and other unspecified comorbid conditions [19]. In our study, we initially hypothesized that allergic (asthma) and non‐allergic (diseases that may be associated with cardiac and pulmonary failure) chronic comorbidities and the use of medications such as beta‐blockers may have a negative impact, particularly in RA and PA cases; however, no significant differences were found in these parameters between the anaphylaxis groups. This may be attributed to the absence of patients with cardiovascular or non‐asthmatic respiratory chronic comorbidities in our study, both of which can impair cardiovascular or respiratory reserve. Additionally, the presence of comorbid asthma did not result in any differences between the groups.

As expected, RA and PA were observed exclusively in severe cases. Hypotension, as a cardiovascular manifestation, played a more prominent role in determining anaphylaxis severity than hypoxia resulting from bronchial or laryngeal obstruction. Similarly, the other studies indicated that RA cases more commonly presented with cardiovascular manifestations, further supporting our findings [17, 18]. However, BA was capable of presenting with all grades of severity, ranging from mild to severe. This finding underscores the importance of administering IM adrenaline even in mild cases. Moreover, our observation that even the second reaction can be severe, consistent with previous studies, further underscores the critical importance of administering adrenaline during the index reaction [10, 11].

In the acute management of anaphylaxis, IM adrenaline remains the first‐line treatment and the one constant across all guidelines for many years. More importantly, our findings suggest that the relatively low rates of IM adrenaline administration (52.3% in Group 1 and 69.2% in Group 2) underscore the need to address persistent gaps in knowledge and clinical practice regarding the emergency management of anaphylaxis. We believe this discrepancy is primarily driven by the failure to administer first rescue treatment in almost half of the BA cases.

This study has several limitations that should be acknowledged. Although the overall number of anaphylaxis cases appeared sufficient, BA, RA, and PA phenotypes were infrequent; therefore, comparisons among these groups could not be undertaken, and findings for these rare phenotypes could only be presented descriptively. Additionally, although the diagnosis of anaphylaxis was reliably established based on objective clinical criteria, serum tryptase measurements were not available for all patients due to the retrospective design of the study. Furthermore, the low proportion of unknown triggers and the relative prominence of drug‐induced anaphylaxis compared with other pediatric cohorts, may reflect selection and documentation bias, as well as the tertiary referral nature of our center.

On the other hand, our database was comprehensive, with minimal missing data, and triggers were confirmed in all FIA and VIA cases and in most DIA cases where diagnostic evaluation was feasible. To our knowledge, this is the first study to concurrently characterize BA, RA, and PA and compare them with conventional anaphylaxis. Future prospective multicenter studies with larger cohorts and systematic biomarker collection, such as serum tryptase measurements, could help to better distinguish phenotypes.

## 5 Conclusion

BA, RA, and PA are rare phenotypes of anaphylaxis that typically present at a later age and are more frequently drug or venom induced. However, these phenotypes may occur at any age, including infancy, and can be triggered by any type of elicitor. It appears that there is still considerable progress to be made in ensuring the appropriate administration of IM adrenaline even in these rare phenotypes.

## Author Contributions

**Gizem Koken:** conceptualization, methodology, software, data curation, formal analysis, investigation, writing – original draft, writing – review and editing. **H. Ilbilge Ertoy Karagol:** conceptualization, methodology, writing – review and editing. **Sinem Polat Terece:** software, data curation. **Ceren Varer Akpinar:** software, data curation. **Kenan Cetin:** software, data curation. **Zeynep Cavdar:** software, data curation. **Berkehan Kara:** software, data curation. **A. Kubra Baskin:** writing – review and editing, methodology, conceptualization. **Arzu Bakirtas:** writing – review and editing, conceptualization, methodology.

## Funding

The authors have nothing to report.

## Conflicts of Interest

The authors declare no conflicts of interest.

## Data Availability

The data that support the findings of this study are available from the corresponding author upon reasonable request.
